# The Neuropsychology of Chronic Neurological Disorders: A Review of Cognitive and Emotional Impairments

**DOI:** 10.7759/cureus.93642

**Published:** 2025-10-01

**Authors:** Harsh S, Haseeb Javaid Rather, Amitabh Dwivedi, Suman Bala, P. Velladurai, Seethalakshmy Anantharaman

**Affiliations:** 1 Department of Forensic Medicine and Toxicology, Teerthanker Mahaveer Medical College and Research Centre, Moradabad, IND; 2 Department of Emergency Medicine, Pt. Madan Mohan Malviya Hospital, New Delhi, IND; 3 Department of Neurology, Shyam Shah Medical College, Rewa, IND; 4 Department of Home Science, Kurukshetra University, Kurukshetra, IND; 5 Postgraduate and Research Department of Zoology, The American College, Madurai, IND; 6 Department of Psychology, Rathinam College of Arts and Science, Coimbatore, IND

**Keywords:** chronic neurological disorders, cognitive impairment, emotional dysregulation, neuropsychological assessment, personalized care, rehabilitation

## Abstract

Chronic neurological disorders (CNDs) represent a broad spectrum of conditions, including Alzheimer’s disease, Parkinson’s disease, epilepsy, multiple sclerosis, stroke, and traumatic brain injury that significantly impair cognitive and emotional functioning. These neuropsychological deficits are not merely ancillary to neurological deterioration but are core features that profoundly affect daily living and long-term outcomes. This review synthesizes current evidence on the cognitive domains most commonly affected, such as memory, attention, executive function, and language, and the spectrum of emotional disturbances, including depression, anxiety, apathy, and mood dysregulation. By examining disorder-specific profiles, neurobiological underpinnings, and brain-behavior relationships, the review underscores the interdependence between cognitive and emotional processes. Further, it evaluates standard and emerging neuropsychological assessment tools, including neuroimaging and digital platforms, and outlines intervention strategies ranging from cognitive rehabilitation and psychotherapy to pharmacological and integrative care models. Limitations in cultural adaptation, technological implementation, and longitudinal tracking are discussed alongside future research directions emphasizing AI, genomics, and personalized medicine. The findings advocate for a multidisciplinary and patient-centered approach to neuropsychological care, which is essential for enhancing quality of life in individuals affected by CNDs.

## Introduction and background

Chronic neurological disorders (CNDs) are a heterogeneous group of progressive or chronic disorders with the feature of impairment of the structure and functioning of the nervous system. These are neurodegenerative diseases like Alzheimer’s disease (AD) and Parkinson’s disease (PD), neurodevelopmental disorders like attention deficit hyperactivity disorder (ADHD), and acquired disorders like traumatic brain injury (TBI), epilepsy, as well as multiple sclerosis (MS). CNDs usually present themselves with a syndrome of motor, sensory, cognitive, and emotional dysfunctions that are progressive, causing significant disability and impaired quality of life [1]. Diagnosis of these disorders usually depends on their cause, the way they develop, and the dominant symptomatology. Others, such as epilepsy and TBI, can be acute but lead to lasting neuropsychological sequelae, whereas others, such as Alzheimer’s and Parkinson’s, take a slow, progressive course of neural degradation [2,3].

One of the most significant and, at the same time, least recognized parts of CNDs is the mental and emotional disability linked with the development of the disease. Cognitive impairments, which include mild memory impairments, moderate executive impairments, and severe executive dysfunction, are also widespread in all CNDs and have been widely reported in conditions like dementia, epilepsy, and post-stroke syndromes [4]. Mental disturbances that include anxiety, depression, apathy, and irritability are also widespread and usually worsen the cognitive dysfunction and hinder rehabilitation processes [5]. These neuropsychological symptoms could precede even the overt neurological symptoms, or last long after the initial injury, or even vary unpredictably as the disease progresses, making diagnosis and management a problem [6].

CNDs are increasingly more common worldwide, mainly due to the aging population, improved survival after acute neurological events, and improved awareness of diagnosis. As an example, dementia is an issue that already affects more than 55 million people globally, and this is an issue that is expected to increase twofold per 20-year period [7]. The increased burden of CNDs is converted into massive personal, social, and economic costs, especially in long-term care costs, productivity loss, and caregiver stress. It is specifically alarming that cases of cognitive impairment in young people are becoming more frequent and are especially caused by sports-related concussion, post-COVID neurological syndromes, and the growing prevalence of developmental conditions like ADHD [8,9].

Neuropsychology is one such interdisciplinary field that has come to be an important study area in light of this increasing challenge, as it integrates neuroscience, psychology, and clinical medicine. In assessing and treating cognitive and emotional dysfunction in people who have neurological conditions, neuropsychologists use structured assessment tools and behavioral interventions. Their use goes as far as early diagnosis and differential diagnosis to planning rehabilitation and observing the outcome of the treatments [10,11]. The use of neuropsychological testing as part of the standard care of patients with epilepsy, MS, and functional neurological disorders (FND) has increased diagnostic specificity and targeting of therapy [12]. Moreover, neuropsychological studies have also given us answers to the mechanisms of the disease (e.g., neuroinflammation as a contributing factor to cognitive disorders, the impact of long-term pharmacotherapy on attention and learning) [13,14].

An increasing awareness of the biopsychosocial model is also evident in the interpretation of CNDs as the psychological, neurological, and environmental factors interact and determine the disease expression and patient experience [15]. This model promotes a comprehensive approach to the care of patients, and is biologically based, but also considers the emotional and social conditions under which symptoms occur or continue. As an illustration, there are a lot of cases of children with neurological developmental disorders that present with overlapping cognitive and emotional symptoms that demand combined assessment frameworks and individual intervention plans [16].

Search and selection methods

This review was conducted using a narrative approach. Relevant peer-reviewed literature was identified through electronic searches in databases such as PubMed, Scopus, and Google Scholar, using combinations of keywords including “chronic neurological disorders,” “neuropsychology,” “cognitive impairment,” and “emotional dysregulation.” Priority was given to recent studies (2015-2024) and high-quality systematic reviews, though seminal earlier works were also considered. Articles were included if they addressed cognitive and/or emotional aspects of chronic neurological conditions such as AD, PD, epilepsy, MS, stroke, or TBI. Exclusion criteria included case reports, non-peer-reviewed sources, and studies not available in English. Reference lists of included papers were also screened to identify additional relevant sources.

## Review

Neuropsychological foundations of cognitive and emotional functioning

Brain-Behavior Relationships in CNDs

To comprehend the neuropsychological bases of CNDs, one needs to discuss the complex relationship between the structure of the brain, cognitive modalities, and emotional regulation. The most important areas relating to cognition and emotion are the frontal lobes (particularly the prefrontal cortex), the temporal lobes, the hippocampus, the amygdala, and the connected components of the limbic system. These areas are selectively impaired in several CNDs, leading to disorder-specific neuropsychological profiles [10].

Role of the Frontal, Temporal, and Limbic Systems

**Figure 1:**
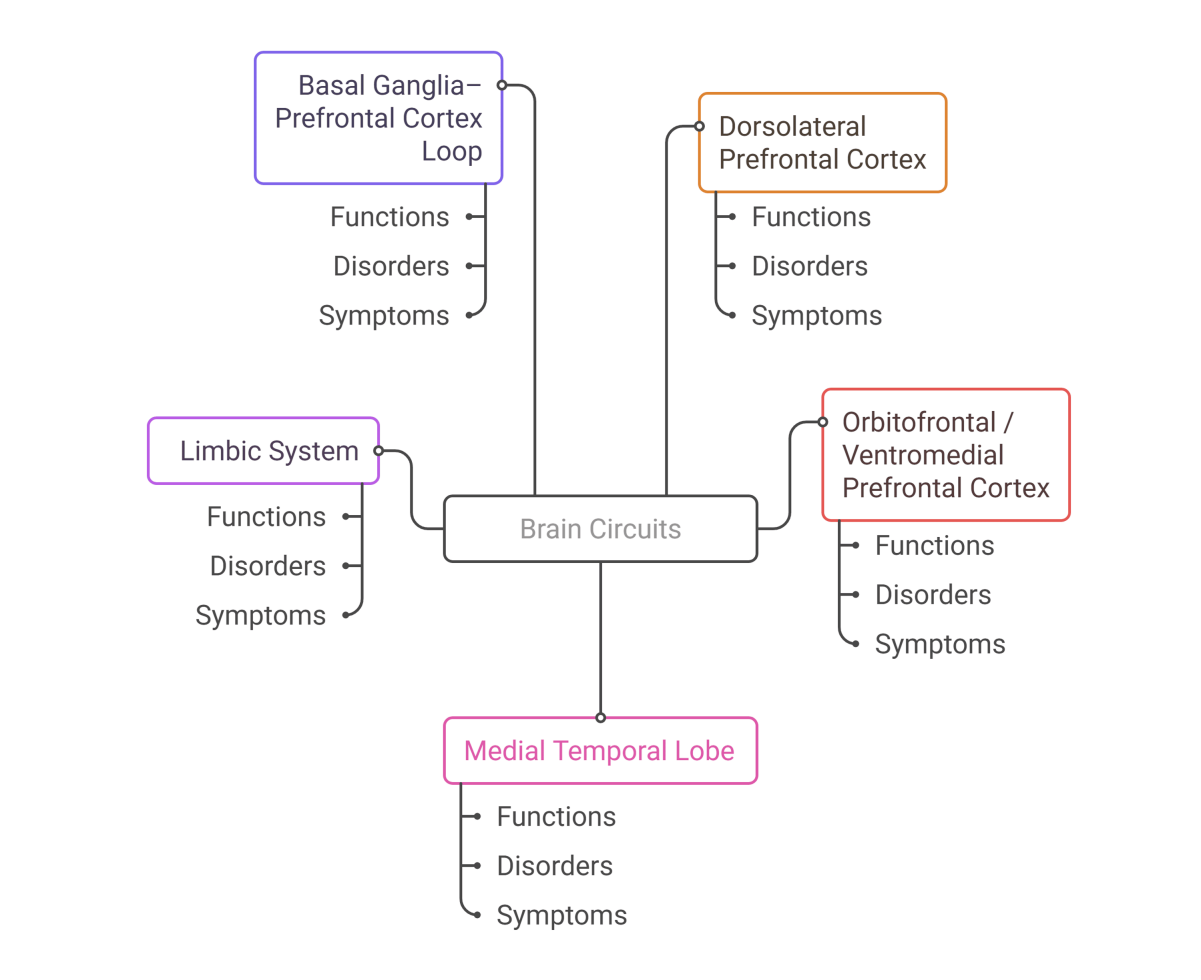
Mindmap of Brain Circuits (Dorsolateral Prefrontal Cortex, Orbitofrontal/Ventromedial Prefrontal Cortex, Medial Temporal Lobe, Limbic System, and Basal Ganglia-Prefrontal Cortex Loop) Image Credit: Harsh S

Higher-order executive functions of working memory, planning, problem-solving, and inhibitory control occur in the frontal lobes, especially the dorsolateral prefrontal cortex. Disorders of these areas are frequent in PD, TBI, and dementia with the frontal variant, which typically include impaired decision-making, perseveration, and lack of insight [11]. Memory consolidation and language comprehension are focused on the temporal lobes, which contain the medial temporal lobe structures such as the entorhinal cortex and the hippocampus. They are highly involved in Alzheimer's disease and temporal lobe epilepsy, in which episodic memory and naming deficits are the most prominent symptoms [12]. The amygdala and the other parts of the limbic system, including the cingulate cortex and hippocampus, are vital structures that process feelings and modulate emotion. Malfunction of such structures is linked with mood disorder, irritability, and emotional lability that are common in MS and post-stroke syndromes [13]. These interrelations of the brain-behavior are central to the preservation of functional autonomy in the sense that disruption of a single node of these circuits interconnected with one another can cause extensive disturbances in thought and emotion. Mindmap representation (Figure 1) of key brain regions (dorsolateral prefrontal cortex, orbitofrontal/ventromedial prefrontal cortex, medial temporal lobe, limbic system, and basal ganglia-prefrontal cortex loop), illustrating their main functions, related clinical disorders, and hallmark symptoms.

Affected Cognitive Domains in CNDs

Neuropsychological impairments manifest across a range of cognitive domains. The most well-known cognitive symptom of CNDs is probably memory dysfunction, which in Alzheimer's disease and chronic traumatic encephalopathy (CTE) begins and worsens early and in both verbal and visuospatial memory [14]. Poor attention and concentration are shared between different disorders, such as ADHD, epilepsy, and MS, and usually result in impairment of academic or professional functioning [15]. Disturbances in language, such as anomia and agrammatism, are also common in stroke-induced aphasia and frontotemporal dementia [16]. Disorders, such as PD and Huntington's disease, are reflected by executive dysfunction, which is an inability to think coherently and make sound judgments, as well as the lack of flexibility of thinking that negatively affects daily decision-making and goal-directed behavior [17].

Interplay Between Emotion and Cognition

Emotion and cognition are deeply intertwined. Systems of emotional processing, such as the amygdala and anterior cingulate cortex, are used to review environmental stimuli and trigger suitable emotional responses. These regions can be damaged in such a way that they dull the emotional reactivity or result in hyperemotionality, depending on the extent and the site of the lesion [18]. Permanent emotional disorders may complicate cognitive defects through disturbance of concentration, encoding of memory, and speed of processing, leading to a cause-and-effect relationship that reduces the chances of functional restoration and integration into society.

Cognitive Reserve and Neuroplasticity

The other two concepts are critical to comprehending variability in clinical outcomes: cognitive reserve and neuroplasticity. The cognitive reserve is the ability of the brain to resist the attacks of neuropathology, which is determined by such factors as education, intellectual activities, and lifestyle. Individuals whose cognitive reserve is higher usually delay or exhibit milder symptoms of pathology despite an equal amount in the brain [19]. The rehabilitation and compensation of chronic neurological illnesses are based on neuroplasticity, which is the ability of the nervous system to reorganize structurally and functionally. Neuroplastic changes may be adaptive or maladaptive and are of special interest in the context of recovery following stroke, TBI, and progressive illnesses such as MS [20].

Cognitive impairments in major CNDs

Alzheimer’s Disease and Other Dementias

AD is the most prevalent form of dementia globally and is an illness that involves deterioration in cognition, which first begins with episodic memory and spreads to other areas, such as language, visuospatial abilities, and executive functions. In neuropathology, AD is linked to amyloid-beta plaques and neurofibrillary tangles, and in particular medial temporal lobes [14]. Three aspects of them, delayed recall, recognition memory, and verbal fluency, are normally underperformed in neuropsychological assessments in AD [21]. Other types of dementia, including frontotemporal dementia and Lewy body dementia, also have different cognitive signatures, e.g., early executive and behavioral dysfunction in the former, visuospatial and attentional deficits in the latter, underlining the importance of a detailed cognitive characterization [22].

Parkinson’s Disease

PD is a neurodegenerative condition whose symptoms are mainly motor, although cognitive problems are now also seen as core pathologic features. Motor decline is frequently preceded or accompanied by executive dysfunction, attentional deficit, and visuospatial impairment [15]. PD is specifically susceptible to subcortical-frontal circuits, resulting in bradyphrenia (slowness of thought), difficulty with set-shifting, and impaired working memory. Cognitive impairment in PD may develop into PD dementia (PDD), with up to 80% of patients developing this in the long run [17]. Planning, abstract reasoning, and verbal fluency difficulties are commonly reported on neuropsychological testing in PD [23].

Multiple Sclerosis

MS is a frequent inflammatory demyelinating disease of the central nervous system that has high rates of cognitive dysfunction. It has been estimated that between 40 and 65% of patients with MS have cognitive deficits, which tend to occur at the early stages of the disease [13]. Typical areas involved are processing speed, attention, working memory, and executive functioning. The heterogeneity of cognitive symptoms is associated with lesion load and atrophy of particular areas, including the thalamus and corpus callosum [11]. More elaborate neuroimaging methods have found relationships between white matter injury and cognitive functioning in MS [24]. One of the weakly acknowledged but highly disabling symptoms is cognitive fatigue, which disrupts prolonged mental activity and daily performance.

Epilepsy

Epileptic cognitive impairment is varied, and it is condition-specific to the etiology, seizure type, age of onset, and medication use. Memory deficits are best linked with temporal lobe epilepsy, and verbal episodic memory in dominant hemisphere foci [12]. Epilepsy in the frontal lobe can cause executive dysfunction, poor control of attention, and problem-solving impairments [25]. Long-term use of antiepileptic drugs, the cumulative consequence of repeated seizures and interictal discharges, may induce more widespread cognitive decline, particularly in treatment-resistant patients [26]. Epilepsy in children also poses another challenge because it affects neurodevelopment and academic performance.

Stroke and Chronic Traumatic Brain Injury

Stroke and the associated long-term impairments are the most common causes of long-term disability in the global community, with as many as 70% of stroke patients developing cognitive impairment [19]. The deficit pattern is very much dependent on the vascular territory involved. Stroke occurring in the left hemisphere usually causes aphasia and loss of verbal memory, whereas left-sided lesions cause neglect, visuospatial dysfunction, and poor awareness [18]. Strokes that affect the anterior cerebral artery or subcortical areas are characterized by executive dysfunction. Cognitive deficits that appear after stroke can last years and are indicative of lower functional independence and quality of life [20].

It is known that chronic TBI, such as repetitive mild TBI in contact sports, is linked to long-term cognitive sequelae and a risk factor of CTE [2,3]. Attention, memory, and speed of processing are common cognitive areas affected. Such persons who experienced numerous concussions are especially at risk of experiencing cumulative neurocognitive deficits, despite lacking clear structural impairment [27]. The deficits in executive functioning and working memory can extend long after recovery, which makes the process of returning to work and social reintegration difficult. There exist other risks associated with pediatric stroke and TBI in that they interfere with the critical developmental stages, as well as brain maturation [10]. Table 1 summarizes the characteristic cognitive and emotional profiles of major CNDs, along with their diagnostic relevance.

**Table 1:** Neuropsychological Profiles in Major Chronic Neurological Disorders

Disorder	Predominant Cognitive Deficits	Emotional Symptoms	Affected Brain Regions	Diagnostic Utility	References
Alzheimer's Disease (AD)	Episodic memory, language, and executive dysfunction	Depression, apathy	Hippocampus, entorhinal cortex, cortex	Early memory loss aids differential dementia diagnosis	[12]
Parkinson’s Disease (PD)	Executive function, attention, and visuospatial skills	Anxiety, depression, emotional blunting	Subcortical-frontal circuits	Helps distinguish PD dementia vs. Lewy body dementia	[17]
Epilepsy (Temporal Lobe Epilepsy (TLE), Frontal Lobe Epilepsy (FLE))	Memory, attention, executive function	Irritability, depression	Temporal/frontal lobes	Guides surgical candidacy and localization	[16]
Multiple Sclerosis (MS)	Processing speed, attention, and working memory	Apathy, mood swings	White matter tracts, thalamus	Correlates with lesion load and MS progression	[13,15]
Traumatic Brain Injury (TBI)	Attention, memory, executive dysfunction	Depression, emotional lability	Frontal lobes, diffuse axonal injury	Aids severity classification and rehab planning	[11,14]
Stroke (Left Hemisphere(LH)/Right Hemisphere(RH))	Language, attention, and visuospatial function	Mood lability, irritability	Hemisphere-dependent vascular lesions	Differentiates aphasia vs. neglect syndromes	[16]
Frontotemporal Dementia (FTD)	Executive function, social cognition	Disinhibition, apathy	Frontal and anterior temporal lobes	Crucial for distinguishing FTD from AD	[17]

Emotional and behavioral dysregulation

Common Emotional Symptoms in CNDs

Depression, anxiety, apathy, and mood lability are all common emotional symptoms reported in patients with CNDs and have a strong impact on the prognosis and quality of life. These emotional expressions are not just secondary responses; they tend to be part and parcel of the neurobiology of the affliction. The neurodegenerative diseases (Parkinson's and Alzheimer's disease) are characterized by the highest rate of depression, with about 50% of patients being affected [15]. Apathy, which is a lack of motivation and goal-directed behavior, is not a part of depression, but it is more common in conditions such as frontotemporal dementia and vascular cognitive impairment [16].

It is common to see anxiety in conjunction with cognitive impairments, and in PD and MS, it can be a precursor or enhancer of executive dysfunction [17]. Irritability and emotional dyscontrol are common symptoms of Gilles de la Tourette syndrome, which are usually confounded with tics and compulsive behaviors as a result of disruption in the action-related neural circuits [18]. According to the impaired frontal-subcortical communication, emotional flattening and disinhibition are also noticed in patients with hydrocephalus [19].

Disorder-Specific Emotional Profiles

Every CND is likely to have a specific emotional profile, which depends on the pattern of the lesion, the progression of the disease, and neurochemical changes. As an example, chronic right hemisphere stroke patients can be characterized by emotional neglect, mood lability, and predisposition to falls secondary to hemispheric affective imbalances [20]. When not treated, post-stroke spasticity leads to chronic frustrations, pain, and social isolation [21]. Inflammatory bowel disease, which is traditionally considered to be a gastrointestinal disease, has some pathophysiological processes in common with CNDs, particularly via the brain-gut axis, which acts on mood and behavior [22].

The best example of emotional dysregulation that looks like neurological dysfunction is FND. Neuropsychologists are the key figures in the process of determining functional symptoms, which are the results of emotional trauma, which might be located in subconscious coping measures [23]. In the case of pediatric populations, neurobehavioral abnormalities, as well as mood disorders and developmental delays, can be caused by an infecting disease, i.e., toxoplasmosis [24].

Psychosocial Consequences and Caregiver Burden

The psychosocial consequences of emotional dysregulation are not just limited to the patient but have a deep impact on the caregiver and family systems. Interpersonal relationships may be greatly affected by emotional blunting, irritability, or aggression, which decreases social functioning. The burden experienced by caregivers of schizophrenic patients is further aggravated by the chronicity, apathy, and lack of insight by the patient, which frustrates therapeutic alliances and compliance [25].

**Figure 2:**
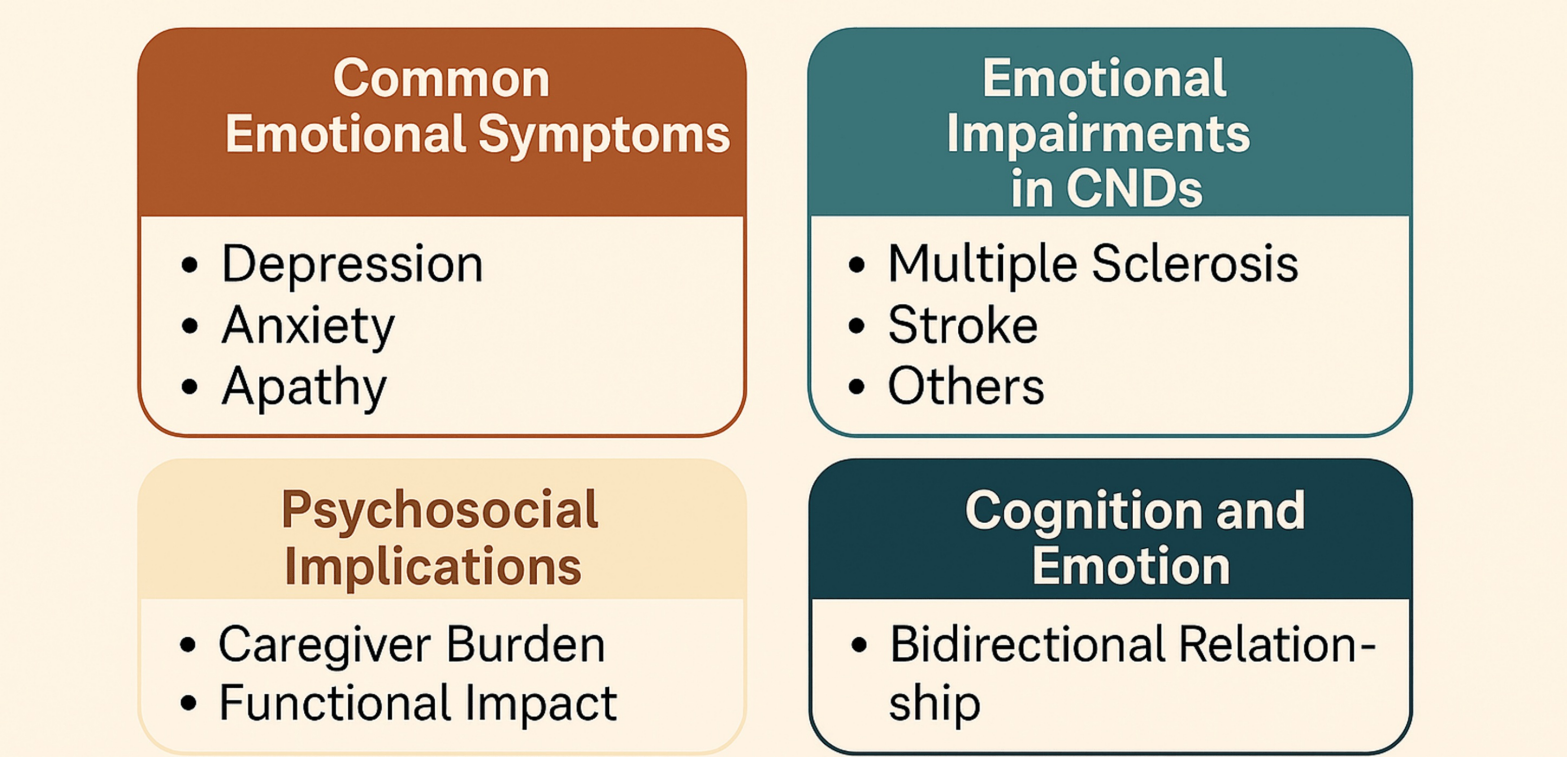
Core Dimensions of Emotional and Behavioral Dysregulation in Chronic Neurological Disorders Image Credit: Harsh S

Children and adolescents with chronic conditions such as fibromyalgia have psychological issues that are distinctive. Their experience of pain is usually not valued, a factor that leads to emotional withdrawal, school absenteeism, and social anxiety [26]. This demonstrates the necessity of early intervention measures that combine emotional treatment with physical rehabilitation. Also, severe emotional flattening is linked to hepatic encephalopathy in old cirrhotic individuals, which leads to the development of exhaustion in the caregivers and institutionalization [15]. As shown in Figure 2, emotional dysregulation in CNDs involves a constellation of symptoms, neuropsychological impairments, and psychosocial consequences that are deeply interrelated.

Cognitive-Emotional Interactions

Emotion and thinking cannot be separated and tend to interact with each other to give rise to intricate symptom complexes. Depression and anxiety are emotional states that worsen working memory, attention, and executive functioning, and cognitive decline increases self-regulation, further increasing the volatility of emotional states [27]. To illustrate, fibromyalgia-related chronic pain changes cognitive appraisal processes and emotional control, which implies the development of integrative models that can combine both sides [27].

Children with biopsychosocial health disruptive diseases, among which are children with developmental neurological disorders, have overlapping emotional and cognitive symptoms that demand combined diagnostic methods [16]. Executive dysfunction is impossible to exist without impulsivity and emotional reactivity in Tourette syndrome, and this aspect underlines the necessity of the broad neuropsychological assessment [18]. Such a cognitive-emotional feedback loop supports the imperative of managing the emotional symptoms not as a subsidiary outcome, but as the fundamental aspects of the disorder.

Assessment tools and diagnostic approaches

Standardized Cognitive and Emotional Batteries

The neuropsychological tests are the foundations of the cognitive and emotional dysfunction diagnosis in CNDs. The Montreal Cognitive Assessment (MoCA) is a typical tool that is commonly applied to identify mild cognitive impairment in numerous conditions, including dementia and hepatic encephalopathy [15]. In executive functioning, tests like the Wisconsin Card Sorting Test (WCST) test mental flexibility, planning, and abstract thinking, which are usually impaired in PD and stroke [16,17].

Emotional assessments are equally crucial. Affective symptoms are measured by the Beck Depression Inventory-II (BDI-II) and the Hospital Anxiety and Depression Scale (HADS), and monitor changes over time [28]. The psychological profiles in pediatric fibromyalgia can be used to differentiate between affective overlays and actual somatic complaints using validated measures [28].

Neuroimaging and Biomarker Innovations

Neuroimaging has revolutionized diagnostic neuropsychology. Volumetric analysis and diffusion tensor imaging (DTI) are all quantitative MRI methods that give information about structural correlates of cognitive deficits. As a case in point, in multifocal sclerosis, thalamic microstructural damage identified through advanced MRI is prognostic of mental performance [29]. Diagnostic enhancement has also been promoted through the development of biomarkers in conditions such as CTE, whose imaging could be insufficient [30].

Imaging and cerebrospinal biomarkers are being considered to be used in the early detection of Alzheimer's, and prismatic adaptation has been demonstrated to be therapeutic in the elimination of spatial and postural deficits after stroke [20]. Although still in their development, these technologies provide potent means of correlating brain pathology with clinical symptoms and disease progression.

Individualized and Longitudinal Assessment

Individualized assessment recognizes the inconsistency in the presentation of the symptoms, disease course, and history of patients. The current approach to the care of stroke in children focuses on the need for multiple assessments with consideration of developmental milestones because initial assessments can overlook delayed or developing deficits [31,32]. Pediatric cognitive profiles, especially epileptic or developmental disorders, are dynamic, and their monitoring is required [33].

Longitudinal cohort-based neuropsychological data indicate that early detection of deficits denotes long-term outcome, which allows planning specific interventions and caregivers [34]. Longitudinal assessment also aids decisions of life regarding autonomy and reintegration in occupations in chronic stroke survivors, as it is used to differentiate between plateau and progressive loss [35].

Integration of Subjective and Objective Measures

Subjective reports and objective testing should be included in a detailed diagnostic model. Most patients, especially epileptics or patients with functional disorders, underreport cognitive or emotional symptoms, depending on a caregiver to confirm such complaints [34]. On the other hand, patients with post-stroke or aphasia can overestimate their improvement, which would require systematic testing to develop correct clinical strategies [35].

Digital technologies give new possibilities for uniting self-reporting with ecological monitoring. The wearable devices and mobile applications have come to the point of providing real-time behavioral data to measure mood swings, cognitive load, and medication compliance. Platforms of telerehabilitation that have already been proven in the visual field training are being customized to cognitive rehabilitation in the case of neurological disorders [36]. These online resources are more accessible, particularly to those patients in rural or low-resource areas.

Equity and cultural adaptation remain pivotal. Instruments that are made in the Western population cannot be taken everywhere without appropriate norming and language validation [36]. Also, sex variation in disease expression, e.g., estrogen-based impacts in epilepsy, plays a role in tool creation and interpretation [37]. Table 2 provides an overview of commonly employed neuropsychological tools used in the assessment of cognitive and emotional dysfunctions across CNDs.

**Table 2:** Commonly Used Neuropsychological Assessment Tools in Chronic Neurological Disorders

Tool/Instrument	Target Domain(s)	Application in Disorders	Notes	Advantages/Limitations	References
Montreal Cognitive Assessment (MoCA)	Global cognition, MCI screening	Alzheimer's disease (AD), Parkinson’s disease (PD), MS	High sensitivity for early-stage deficits	Quick, but lower specificity in low-education groups	[27]
Wisconsin Card Sorting Test (WCST)	Executive function, mental flexibility	Traumatic brain injury (TBI), PD, frontotemporal dementia (FTD)	Assesses frontal lobe function	Sensitive to frontal dysfunction, but culturally biased	[30]
Beck Depression Inventory-II (BDI-II)	Depression severity	Across chronic neurological disorder (CNDs)	Self-report, widely used	Easy to administer, may conflate somatic symptoms	[28]
Hospital Anxiety and Depression Scale (HADS)	Anxiety and depression screening	Stroke, MS, chronic pain	Useful in hospital settings	Good for the medically ill, less comprehensive	[28]
Diffusion Tensor Imaging (DTI)	White matter integrity	MS, TBI	Identifies microstructural damage	High sensitivity, but expensive and complex	[35]
Functional MRI (fMRI)	Brain activation patterns	AD, epilepsy	Useful in mapping brain-behavior links	High spatial resolution, limited accessibility	[25]
Caregiver Burden Scale	Psychosocial burden	Dementia, FTD	Measures the impact on caregivers	Essential for caregiver-targeted interventions	[28]
Telerehabilitation Platforms	Real-time cognitive/emotional training	Stroke, TBI	Accessible for remote care	Promising, but requires digital literacy and access	[38]

The neuropsychologist should be alert to the more subtle psychiatric symptoms that are presented with cognitive deterioration in neurodegenerative and psychiatric overlap syndromes such as traumatic encephalopathy syndrome (TES) [36,38]. Multifaceted assessments are useful in early diagnosis and the distinction of overlapping symptoms. Lastly, assessments and interventions, particularly on the caregiver level, have proven that enhanced adherence to monitoring procedures improves the results in dementia care and lowers burnout levels in the caregivers [39].

Management and intervention strategies

Cognitive Rehabilitation and Training Programs

Cognitive rehabilitation is concerned with the training of particular neuropsychological deficits using specific activities, compensation, and environmental modifications. It has been effective in treating cognitive disorders in various CNDs such as TBI, stroke, and dementia. Cognitive rehabilitation is used in TES to treat deficits in attention, executive dysfunction, and memory loss following repetitive head injury [36]. Programming considerations tend to focus on repetition, goal-setting, and feedback, which facilitate neuroplastic adaptation and functional recovery. Another approach to cognitive enhancement is experimental, so so-called neural grafts in Huntington’s disease. Whereas clinical trials have brought varied results, they demonstrate the promise of biologically mediated cognitive restoration [37]. Still, cognitive rehabilitation is one of the key solutions to the problem, particularly at post-acute stages where there are either no pharmacological alternatives or the ones available are not effective.

Psychological Therapies: Cognitive Behavioral Therapy (CBT), Acceptance and Commitment Therapy (ACT), and Mindfulness

CBT, ACT, and mindfulness-based cognitive therapy (MBCT) are important psychological interventions to deal with the emotional and behavioral aspects of CNDs. Besides reducing depressive and anxiety symptoms, these modalities also increase cognitive flexibility and coping strategies. As an example, psychiatric manifestation precedes cognitive manifestation of CTE and is sensitive to structured psychotherapy [36]. Use of CBT in treatment regimens of neurodegenerative and neuropsychiatric conditions enhances compliance and lowers the burden on caregivers. At the same time, ACT helps to achieve psychological flexibility in patients with progressive illnesses (MS or the initial stages of dementia). These treatments are more critical in cases where a condition such as PTSD, as seen in veterans and athletes with CTE, compromises emotional control [38].

Pharmacological Approaches Targeting Neuropsychological Symptoms

The neuropsychological symptoms can be pharmacologically controlled using antidepressants, anxiolytics, mood stabilizers, and cognitive enhancers. The choice to retire or to keep playing for athletes with frequent concussions is partially dependent on the management of symptoms using pharmacological solutions [39]. Prescriptions of selective serotonin reuptake inhibitors (SSRIs) and those of norepinephrine reuptake inhibitors are common in treating depression and anxiety related to PD, stroke, and epilepsy.

Botulinum toxin has also been used in other ways not related to spasticity management in post-stroke patients, as it has also been found to improve mood and mobility indirectly as a result of improved motor ability [21]. In chronic subdural hematoma, psychopharmacological treatment can be applied as supplemental treatment to stabilize the mood and cognition in the process of post-operative recovery [40]. Nevertheless, these pharmacologic therapies must be closely observed at all times, particularly among older adult patients who exhibit impaired pharmacokinetics and polypharmacy.

Integrative, Multidisciplinary, and Patient-Centered Care Models

Management of CNDs is increasingly becoming multidisciplinary, involving the combination of neurology, psychiatry, neuropsychology, physiotherapy, and social work. These patient-based models seek to care about the individual as a whole as opposed to focusing on individual symptoms. As an example, in chronic migraine, the combination of pharmacological and behavioral therapy and biofeedback has had improved results as compared to monotherapy [41]. Such technological advancements as telerehabilitation promote this model of integration by providing remote or underserved areas with access to care. Recent findings indicate that multisensory training through telerehabilitation is practicable and efficacious in visual field defects and has implications for greater cognitive treatments [42]. Such a strategy is consistent with the trend of a personalized approach to medicine, where the treatment is specific to both the disease and the psychological characteristics of the person, cultural background, and available resources in the environment.

Limitations and future work

Cultural and Diagnostic Standardization Challenges

One of the greatest constraints of the present-day neuropsychological practice is that there exists no cross-cultural standardization of diagnostic instruments. The majority of cognitive and emotional assessment batteries are created within Western contexts and do not consider the linguistic diversity, educational variance, and cultural standards existing in non-Western populations. In some countries, such as India, such shortcomings can lead to misdiagnosis or failure to diagnose people with CNDs. There are attempts to develop neurological disability rules that would be adapted to a particular sociolinguistic environment [43], but there is still no comprehensive validation. Other disorders, such as Wernicke-Korsakoff syndrome, which is commonly misinterpreted because of its cultural misrepresentation in substance use, emphasize the need to have diagnostic clarity and cultural contextualization [44].

Gaps in Longitudinal and Functional Outcome Research

The majority of neuropsychological tests are constrained to a singular point in time, providing only a snapshot of cognitive and emotional functioning. This deficiency of longitudinal data restricts the capability to learn about the improvement of symptoms, recovery trends, and efficiency of treatment in CNDs. Longitudinal studies are specifically important in establishing time windows of early intervention and predictive modeling of long-term outcomes [45]. Additionally, caregiver-mediated interventions benefit significantly from ongoing support structures. The meta-analyses revealed that the long-term online support of the caregivers of dementia patients enhances adherence and decreases psychological burnout, which makes it possible to consider the scalable frameworks that may be applied to other chronic conditions [46].

Integration Barriers in Technological Innovation

The emergence of artificial intelligence (AI) and digital neuropsychology is thrilling and, at the same time, poses the challenges of implementation. The AI-based solutions allow real-time evaluation of cognitive performance, attention, and mood, which increases the ecological validity of tests. Under development, they are meant to detect early symptoms of cognitive deterioration and PTSD among people with TBI [47]. Nonetheless, current clinical integration is not sufficient because of data privacy concerns, cost-related issues, regulatory approval, and technological access inequality. A similar tension exists in biomarker and genomic profiling. Genetic risk stratification is still developing, particularly in epilepsy and Alzheimer's disease, but at this point, these tools are not widely available or fully understood in the current clinical framework [44,45].

Need for Personalized and Translational Approaches

Lastly, CNDs have a high level of heterogeneity, which is a major obstacle to universal treatment plans. The symptoms of emotional, cognitive, and behavioral dysfunctions are usually combined and differ in each person, and, therefore, neuropsychological interventions should be individualized. A gap also exists between research and practice, which must be closed by translational neuroscience, the application of findings in laboratory genomics, imaging, and behavioral science to the management of actual patients [48]. These methods have to be inclusive, affordable, and scalable, such that innovation is made accessible to a large, diverse patient population.

## Conclusions

Chronic neurological diseases are complex and go much beyond motor or sensory symptoms. This review has brought out the neuropsychological complexity of these disorders in that cognitive and emotional impairments are inherent, ubiquitous, and even in some cases progressive. Most of these deficits are supported by core brain areas that include the prefrontal cortex, the limbic system, and the hippocampus, and they are characterized by executive dysfunction, memory loss, language disturbances, mood lability, and behavioral alterations. Such impairments differ according to the disorder and always interfere with the functional autonomy and psychosocial well-being of patients.

The neuropsychological science has been able to provide vital instruments of precise diagnosis, efficient intervention, as well as enhanced prognostic planning through incorporation in clinical neurology. Whether a cognitive rehabilitation approach, psychotherapy approach, neuroimaging innovation, or digital approaches in neuropsychology, interdisciplinary approaches are becoming more common in allowing clinicians to treat patients as a whole. Also, uniform assessment batteries and culturally sensitive diagnostic measures are enhancing accuracy as well as accessibility in neuropsychological care. Neuropsychological services should be incorporated into the standard care model as the load of CNDs keeps increasing worldwide. In addition to improving the cognitive and emotional quality of life of patients, this will aid the caregivers, limit the long-term cost of healthcare, and enable personalized medicine. To conclude, neuropsychological care is not an accessory but a basic component of the overall treatment of chronic neurological diseases.
